# Pathophysiological Factors in the Relationship between Chronological Age and Calculated Lung Age as Detected in a Screening Setting in Community-Dwelling Subjects

**DOI:** 10.3389/fmed.2016.00002

**Published:** 2016-02-03

**Authors:** Peter Kardos, Tanja Schütt, Tobias Mück, Helmut Schumacher, Martin C. Michel

**Affiliations:** ^1^Group Practice, Center for Allergy, Respiratory and Sleep Medicine, Red Cross Maingau Hospital, Frankfurt am Main, Germany; ^2^Department of Medical Affairs, Boehringer Ingelheim Pharma GmbH & Co KG, Ingelheim, Germany; ^3^Statistical Consultant, Ingelheim, Germany; ^4^Department of Translational Medicine and Clinical Pharmacology, Boehringer Ingelheim Pharma GmbH & Co KG, Ingelheim, Germany; ^5^Department of Pharmacology, Johannes Gutenberg University, Mainz, Germany

**Keywords:** COPD, dyspnea, lung age, pathophysiology, spirometry

## Abstract

**Aim:**

To explore the relationship between pathophysiological factors and premature lung aging in a cohort of community-dwelling subjects in a health-screening setting.

**Methods:**

16,107 pharmacy customers in Germany (5954 males, 10,153 females; mean age 59.7 years) participated in a lung function screening project by providing demographic data, including smoking status and known airway conditions and performing spirometry with a Vitalograph, a spirometry screening device. Lung age was calculated from the spirometric findings, and the difference between chronological age and calculated lung age was analyzed in its relationship to the demographic data in general linear models.

**Results:**

In the overall cohort, calculated lung age exceeded chronological age by 10.0 years. Based on the subset of non-smokers not reporting any airway conditions, Vitalograph data in this setting may underestimate FEV_1_ to some degree, but this apparently had little impact on the detection of association of lung age with pathophysiological factors or the corresponding effect sizes. The most important factors associated with greater lung age based on strength of association were presence of dyspnea, being a smoker, and reporting a history of COPD or asthma. Corresponding effect sizes for the difference between age and lung age were 6.5, 5.7, 13.9, and 8.3 years over the chronological age.

**Discussion and Conclusion:**

These data confirm the usefulness of screening devices of lung function testing for epidemiological but potentially also for pharmaco-epidemiological studies.

## Introduction

We all age, and that is not bad at all because there would be no life without aging. Lung function physiologically declines with aging, but only accelerated aging of the lung, due to inhalation of noxious substances, such as smoking, is bothersome and can lead to morbidity and incapability. Aging is inevitable, but healthy aging with maintenance of a good function can be expected. This requires understanding of the aging process and removal of factors associated with and perhaps even causing unhealthy aging, i.e., premature or accelerated functional decline.

Similar to other organs, the lung also exhibits age-associated changes (1). These include a reduced lung volume, ciliary dysfunction, and alterations of the respiratory muscles, leading to a reduced pulmonary efficacy and cough strength. Underlying physiological processes also include complex changes in immunity (1), increased extracellular matrix deposition (2), and an altered responsiveness of the receptor systems regulating lung function (3, 4). Moreover, the senescent lung is also more susceptible to injury as documented in many animal models (5), potentially creating a vicious cycle of age-associated functional impairment and even greater additional damage upon injury.

Clinical studies of age-related changes in pulmonary function are often based on key spirometric parameters, such as forced expiratory volume in 1 s (FEV_1_) and forced vital capacity (FVC); forced expiratory volume in 6 s (FEV_6_) is a suitable alternative to FVC in studies of lung function (6, 7). Therefore, other than symptom assessment (8), changes in FVC, FEV_1_, and FEV_6_ have often been used to study the physiological decline of lung function with advancing age and how this is affected by pathological conditions.

The physiological relationship between age, height, gender, and ethnicity, on the one hand, and lung function, on the other hand, was described in healthy subjects in several sets of reference equations, e.g., European Community for Steel and Coal (ECSC) (9), National Health and Nutrition Examination Survey (NHANES) III (10), and most recently and most comprehensively, the Global Lung Initiative (GLI) 2012 (11). GLI reference equations are available both for children and adults up to 95 years old, for four different ethnicities. Of note, GLI reference equations describe a greater respiratory capacity for a given age than ECSC equations.

The decline in lung function with aging is greater than expected in some patients, which has led to the concept of “lung age.” Usually, the observed FEV_1_ will be related to that for gender, ethnicity, chronological age, and height-matched predicted value (“% predicted”). By contrast, lung age relates the observed FEV_1_ to the age for which the measured value equals 100% predicted. A low observed FEV_1_ of a 48-year-old man (e.g., 2.85 L, corresponding to only 75% of age matching predicted mean) will be now matched to a corresponding higher age (e.g., 2.85 L equals 100% for a 71-year-old man). It is assumed that premature lung aging has occurred due to pathophysiological factors. For instance, increased lung age is observed in patients with COPD (12). Other than providing potentially interesting information on pulmonary pathology, it has been proposed that determination of lung age may be an educational tool, e.g., help to motivate smoking cessation (13, 14). The concept of organ age as an indicator of organ-specific premature aging has also been established for the heart, where it also has been suggested as an educational tool to motivate healthier life styles (15).

The Vitalograph copd-6 spirometer is a portable electronic device primarily developed for screening purposes, particularly with regard to COPD (16). It measures FEV_1_ and FEV_6_ based on forced expiration and, with additional input of gender and height, calculates lung age based on the gender-specific ECSC equations (9). The positive and negative predictive values of detecting airway obstruction with the Vitalograph have been documented (17). The protocol of a validation study comparing Vitalograph data to those of conventional spirometry (18) and reports of a small-scale screening project have been published (19).

Against this background, the present study was designed to screen a large number of community-dwelling pharmacy customers and determine their lung age. We then related the difference between lung age and chronological age to various known pathophysiological factors to explore the usefulness of the Vitalograph, for instance, for future use as an easily understandable and popular smoking cessation aid and/or support of pharmaco-epidemiological studies.

## Materials and Methods

Ethical committee approval was neither required nor recommended for this type of study in Germany at the time it was performed. Similarly, the lung function testing offered to the general public during the 2014 congress of the European Respiratory Society held in Munich, Germany^1^ also did not involve ethical committee approval.

Between November 1 2013 and January 31 2014, 704 German pharmacies were invited to participate in the study. Each pharmacy was supplied with case record forms and a Vitalograph copd-6 spirometer (Vitalograph GmbH, Hamburg, Germany) and received basic training in its use. During the study period, each pharmacy was requested to offer each customer participation in the study. Study participation was voluntary and anonymous and included both a lung function test on the Vitalograph and completing a case record form. This included subject gender, chronological age, height, population size of home town (< vs. >100,000 inhabitants), presence of cough (no, yes; if yes, acute or chronic and with or without expectoration), presence of dyspnea (no, yes; if yes, during usual daily activities or paroxysmal), smoking habit (no, yes; if yes, duration of up to or more than 10 years and frequency of incidentally, 1–10, 11–20, 21–30, and >30 cigarettes per day), presence of allergies (no, yes), and presence of current airway conditions (none, common cold, COPD, asthma, and “other”). For information of the pharmacy and the participant, the case record form provided operational definitions related to cough and dyspnea (Table 1).

**Table 1:** **Operational definitions related to cough and dyspnea provided to pharmacy and study participants on the case record forms**.

–“Chronic” cough: “recurrent (multiple times per month) or regularly or continuously (almost daily for at least 8 weeks)”
–“Dyspnea”: “difficulty of deep inhalation, heavy breathing or rapid exhalation”
o“Paroxysmal”: “only in specific situations [e.g., stress, sport, allergens (pollen, house dust, animal hair)]”
o“Usual daily activity”: “e.g., when climbing stairs”

FEV_1_, FEV_6_, and calculated lung age as determined by the Vitalograph measurement were to be entered into the case record form. The Vitalograph calculates lung age for men and women based on equations provided by Morris and Temple (13), which after conversion to metric units are

Lung age=(113•height)−(31.250•observed FEV1)−39.375 (males)

Lung age=(140•height)−(40.000•observed FEV1)−77.280 (females).

The manufacturer reports the accuracy of the Vitalograph to be <±3%.

In most cases, FEV_1_ and FEV_6_ were entered into the case record forms as absolute value; however, in some cases, FEV_1_ was given as percentage of predicted value (FEV_1__%-predicted), a variable also provided by the Vitalograph. These percentage values were converted into absolute values based on the following equations, which are used by the Vitalograph to calculate the percentage values:

FEV1=(3.62•height−0.0320•chronological age−1.26)  •FEV1_%−predicted (males)

FEV1=(3.50•height−0.0250•chronological age−1.93)  •FEV1_%−predicted (females)

In these equations, FEV_1_ is expressed in liters, height in meters, and chronological age in years.

**Figure 1:**
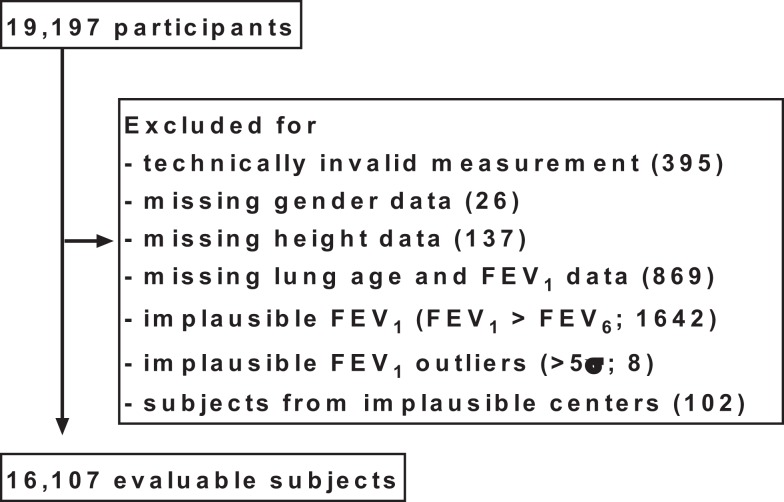
**Disposition of participants**. For some subjects, more than one exclusion criterion applied; for details, see Section “Materials and Methods.”

All analyses described here were specified after completion of the study, but before the case record forms were entered into the database and before start of any analysis. In total, 19,197 subjects aged 20 years or older participated in the study. Subjects were excluded from the analysis if at least one of the following predefined criteria applied to them (Figure 1):

– Vitalograph reading of “!,” indicating a technically invalid measurement (*n* = 395).
– Data missing for gender (*n* = 26).
– Data missing for height (*n* = 137).
– Data missing for lung age and FEV_1_ in case record form (*n* = 869).
– Reported FEV_1_ > reported FEV_6_ in case record form (*n* = 1642).
– Reported FEV_1_ value implausible according to gender-specific 5σ-rule (*n* = 8).
– Subjects from four pharmacies with implausible values, identified by funnel plot (*n* = 102).

Some subjects were excluded due to more than one criterion.

For the remaining subjects (*n* = 16,107), the following adjustments were made in the database prior to any analysis:

– If no response was given for presence of cough (*n* = 124), smoking (*n* = 115), allergy (*n* = 418), common cold (*n* = 13,921), COPD (*n* = 15,724), asthma (*n* = 15,227), dyspnea (*n* = 471), or other airway disease (*n* = 15,378), this was considered as absence of the condition. For some subjects, this applied to more than one of these variables.
– In a small number of cases, “no” was indicated for presence of cough but positive statements on duration or expectoration were provided (*n* = 5), or “no” was indicated for smoking but information on number of cigarettes or duration of smoking status was given (*n* = 5), or “no” was indicated for dyspnea but information on frequency of dyspnea was given (*n* = 7); in those cases, the “no” was reset to “yes” for plausibility reasons.

All analyses were performed using 16,107 subjects. In line with the *post hoc* character of the analyses, all statistical analyses are descriptive and exploratory. Two-sided *P*-values below 0.01 were interpreted as indicating a significant effect. Data analysis was performed using SAS (version 9.4, SAS Institute Inc., Cary, NC, USA).

## Results

### Description of Study Population

After application of the predefined exclusion criteria, data from 16,107 subjects (37.0% men; 28.9% living in large city) were available for analysis. Their demographic and airway-specific data are summarized in Table 2. Participants had a mean chronological age of 59.7 years but covered a wide range (20–98 years). 21.2% of participants self-identified as smokers, and 16.5% reported being a smoker for more than 10 years, mostly reporting not more than 20 cigarettes per day. Men as compared to women were slightly older, more likely to be a smoker and, if a smoker, tended to smoke more cigarettes per day (Table 2).

**Table 2:** **Demographic and airway-specific data in the total study group and in subgroups of male and female participants**.

	Total	Males	Females
**Demographics**			
Number	16,107	5954	10,153
Age, years	59.7 ± 16.4	61.3 ± 16.5	58.7 ± 16.2
Height, centimeter	168.9 ± 9.1	176.6 ± 7.3	164.4 ± 6.7
Living in large town, %	28.9	29.7	28.4
Smoker, %	21.2	23.3	20.0
Smoker for more than 10 years, %	16.5	18.4	15.4
Smoker with 1–10/ 11–20/21–30/>30 cigarettes/day, %	7.6/8.3/3.0/0.8	6,9/9.5/3.9/1.5	8.0/7.6/2.4/0.4
**Patient symptoms and disease history**			
Cough, %	33.8	32.6	34.4
Cough with expectorations, %	18.3	19.2	17.7
Chronic cough, %	17.5	16.0	18.3
Dyspnea, %	31.6	24.6	35.6
During usual daily activities, %	16.3	11.8	18.9
Patient-reported allergies, %	26.0	18.9	30.1
Common cold, %	13.6	13.4	13.7
COPD, %	2.4	2.8	2.1
Asthma, %	5.5	4.0	6.3
Other airway disease, %	4.5	4.0	4.8
**Airway parameters**			
FEV_1_, L	2.33 ± 0.86	2.78 ± 0.94	2.07 ± 0.69
FEV_1_, % predicted	87.5 ± 22.6	87.5 ± 24.4	87.6 ± 21.5
FEV_6_, L (*n* = 11,574/4274/7300)	2.91 ± 1.01	3.50 ± 1.10	2.57 ± 0.78
Lung age, years	69.8 ± 24.3	71.0 ± 25.0	69.0 ± 23.8
Difference lung age – chronological age, years	10.0 ± 18.9	9.5 ± 19.8	10.2 ± 18.3

*Data are means ± SD or percentage of respective group*.

Cough was reported in 33.8% of the study population, and more than half of those reported chronic cough and/or cough with expectorations (Table 2). Dyspnea, allergies, common cold, COPD, asthma, and other airway diseases were reported in 31.6, 26.0, 13.6, 2.4, 5.5, and 4.5%, respectively. As compared to women, men reported cough less frequently but more frequently cough with expectorations. Notably, men reported dyspnea and allergies much less frequently than women (Table 2).

The mean measured FEV_1_ and FEV_6_ was 2.33 and 2.91 L, respectively, with men, as expected, exhibiting larger values than women (Table 2). Calculated lung age was 69.8 years in the total population, which was 10.0 years greater than chronological age; this difference was smaller in men than in women (9.5 vs. 10.2 years, Table 2).

### Method Validation

Considering that Vitalograph measurements in a screening setting with limited training of pharmacy personnel may lead to falsely low estimates of FEV_1_ due to submaximal effort in forced expiration, we have used the group of non-smokers not reporting any type of airway disease with documented FEV_1_ values (1521 males, 2006 females) to determine the linear relationship of FEV_1_ to height and chronological age in our dataset. This was done by the Global Linear Model procedure in SAS. The resulting coefficients in the equations are similar to those reported by Morris and Temple (13). They were as follows (with 95% CI for estimates of coefficients):

$$\begin{array}{l} {\text{FEV}}_{\text{1}}=\text{3}.\text{25}•\text{height}-0.0\text{3}0\text{2}•\text{chronological age}-0.\text{973 }\left(\text{males}\right)\\ \;(\text{2}.\text{76}-\text{3}.\text{75})\;(0.0\text{277}-0.0\text{333})\;(-0.0\text{31}-\text{1}.\text{91}) \end{array}$$

$$\begin{array}{l} {\text{FEV}}_{\text{1}}=\text{2}.\text{99}•\text{height}-0.0243•\text{chronological age}-1.31\;\left(\text{females}\right)\\ \;(\text{2}.\text{66}-\text{3}.\text{33})\;(0.0\text{230}-0.0\text{257})\;(0.732-\text{1}.\text{89}) \end{array}$$

### Analyses Using Multiple Linear Models

**Figure 2:**
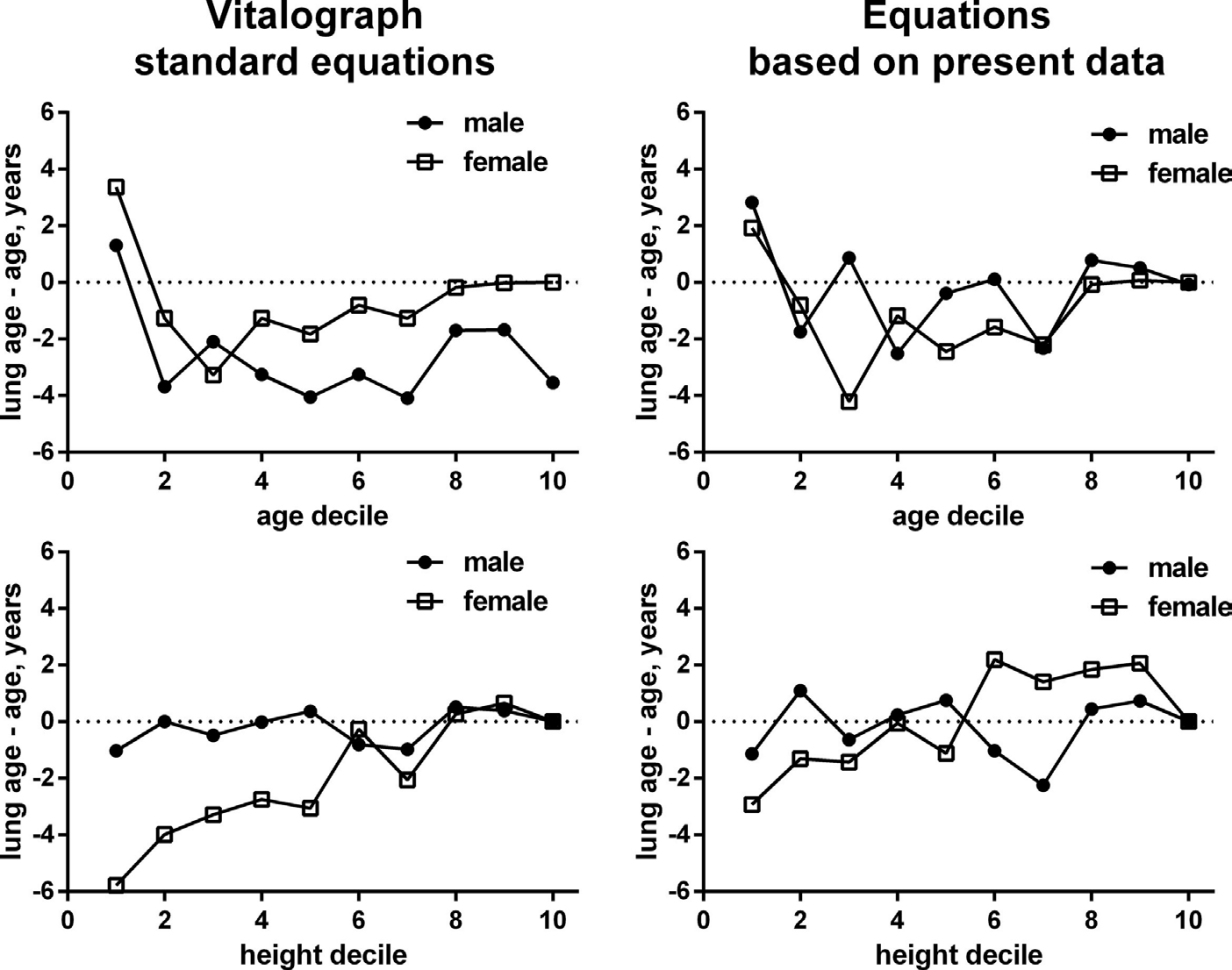
**Gender-specific associations between gender and chronological age (upper panels) and height (lower panels) for the difference between lung age and chronological age**. Numbers in the left panels are based on the ECSC equations build into the Vitalograph, numbers in the right panels based on the empirically derived equations in non-smokers not reporting any airway condition. The scale of the *x*-axis represents deciles of male or female subjects for age and height. Each decile includes the data from 10% of subjects in ascending order of age or height, respectively, see Table 3. Note that the depiction based on our empirical equations may in part reflect a self-fulfilling prophecy as some of the data went into the derivation of the equation and the depicted data.

Taking into account the large inter-individual variation of the difference between lung age and chronological age, we have applied general linear model methodology to explore factors associated with an older lung age. The model selection procedure included gender, chronological age, height, city size, smoking status, and presence of cough, common cold, allergies, dyspnea, COPD, asthma, and “other respiratory disease” as potential explanatory variables (Table 3). The stepwise selection process applied parameter-specific P-limits of 0.25 for entry into the model and of 0.15 for stay in the model. As we empirically found a different equation to better describe chronological age-, height-, and gender-specific FEV1 in non-smokers not reporting any airway disease (see previous paragraph), the models were applied using the equations used by the Vitalograph and those empirically determined by us. Both models yielded very similar information regarding the factors identified as being possibly associated with the difference between chronological age and lung age. The strongest association (based on F-statistic) existed for dyspnea with a parameter estimate of 6.5 and 5.8 in the two models; this means, that with all other factors being equal subjects with dyspnea had an additional 6.5 or 5.8 years greater difference between lung age and chronological age if compared to subjects without dyspnea. Other parameters associated with an older lung age in descending order of strength of association were: being a smoker, presence of COPD, presence of asthma, chronological age and height decile, presence of cough, presence of “other airway disease,” and presence of common cold; presence of allergies did not consistently exhibit a significant association across the two models, possibly because it was a weak relationship even in the model that detected it as significant. Of note, the association between lung age and chronological age or height may not be strictly linear (Figure 2).

**Table 3:** **Association of potential explanatory variables with a greater difference between lung age and chronological age**.

	Vitalograph standard equation		Equation based on present data	

	*P*-value	Change in lung age ± SE	*P*-value	Change in lung age ± SE
Dyspnea	<0.0001	6.5 ± 0.32	<0.0001	5.8 ± 0.47
Smoker	<0.0001	5.7 ± 0.37	<0.0001	6.3 ± 0.53
COPD	<0.0001	13.9 ± 0.94	<0.0001	14.1 ± 1.30
Asthma	<0.0001	8.3 ± 0.64	<0.0001	8.8 ± 0.91
Chronological age decile	<0.0001	See Figure 2	<0.0001	See Figure 2
Height decile	<0.0001	See Figure 2	<0.0001	See Figure 2
Cough	<0.0001	1.7 ± 0.34	<0.0001	2.0 ± 0.49
Other airway disease	<0.0001	3.2 ± 0.69	0.0021	3.1 ± 1.00
Common cold	0.0006	1.5 ± 0.45	0.0048	1.8 ± 0.65
Patient-reported allergies	0.0052	−0.9 ± 0.33	0.21	−0.7 ± 0.46

*Analyses were performed based on the equations used by the Vitalograph (“Vitalograph standard equation”) and the equation we have empirically derived in the subset of non-smokers not reporting any airway disease in this study (“equation based on present data”). Results are based on a selection procedure to identify predictors of high lung age using a multivariate linear model and shown as parameter estimates with SE and corresponding *p*-value. Parameter estimates are displayed in the sequence of inclusion into the model (based on F-tests). For categorical variables effects are relative to the respective control group of non-smoker, and absence of cough, allergies, COPD, asthma, dyspnea, or other airway disease. Note that the parameter estimates for chronological age and height are given for gender-specific deciles, respectively. Size of home town is not included because it did not reach the predefined threshold level of *p* < 0.15 for staying in the model. The parameter estimate for change in lung age is expressed in years*.

To further explore the impact of additional information on dyspnea, smoking, and cough, the same linear models were applied to the subsets of participants with the respective characteristic (Table 4) and available additional information. In the subset of subjects with dyspnea (*n* = 4773), subjects reporting it during usual daily activities by average had a 5.0 years older lung age than those with paroxysmal dyspnea. While smokers had a greater lung age than non-smokers (Table 3), a dose–response relationship for number of cigarettes smoked per day and difference between lung age and chronological age was observed among the 3190 smokers; parameter estimates for the difference between lung age and chronological age increased from 6.1 in those consuming up to 10 cigarettes per day to 15.4 in those smoking more than 30 cigarettes per day as compared to those reporting occasional smoking only (Table 4). However, duration of smoking habit for >10 vs. ≤10 years did not exhibit a significant contribution. Finally, in the subset of participants with cough (*n* = 4895) neither presence of expectoration nor chronic vs. acute cough had significant contributions.

**Table 4:** **Association of additional explanatory variables with a greater lung age in the subsets of subjects with dyspnea, smokers, and subjects with cough and respective additional information**.

	Change in lung age **±** SE	*p*-value
**Subset of subjects with dyspnea (*n*** **= 4773)**		
Dyspnea during usual daily activities vs. paroxysmal dyspnea	5.0 ± 0.56	<0.0001
**Subset of smokers (*n*** **= 3190)**		
1–10 cigarettes/day	6.1 ± 1.92	0.0016
11–20 cigarettes/day	9.2 ± 1.92	<0.0001
21–30 cigarettes/day	12.2 ± 2.07	<0.0001
>30 cigarettes/day vs. occasional smoking	15.4 ± 2.61	<0.0001
Duration of smoking >10 years vs. ≤10 years	−1.2 ± 1.03	0.25
**Subset of subjects with cough (*n*** **= 4895)**		
Expectoration vs. no expectoration	1.0 ± 0.56	0.072
Chronic vs. acute	−0.7 ± 0.60	0.22

*These analyses were performed based on the equations built into the Vitalograph. The parameter estimate for change in lung age is expressed in years*.

## Discussion

The present study demonstrates a difference of 10 years between lung age and chronological age in a large cohort of community-dwelling subjects. This is even more remarkable as the calculation of lung age by the Vitalograph is based on the ECSC equations (9), which, relative to the GLI 2012 equations (11), underestimate predicted FEV_1_. Accordingly, the difference between lung age and chronological age may be even larger than found here.

### Limitation of Methods

The current study is based on community-dwelling subjects visiting a pharmacy for any reason. While the participating pharmacies were requested to offer study participation to each customer, it is possible that subjects concerned about their lung function may have been more likely to participate than those without. Thus, the pharmacy setting implies that our cohort despite being population based may be biased toward people having health complaints. This possible selection bias needs to be taken into consideration in the interpretation of both the demographic and the lung function data. However, we consider this unlikely to have limited our regression analysis to determine factors associated with lung age as it affects the distribution between healthy and diseased participants as compared to a population-based cohort but should have limited effect on the association between pathophysiology and lung age.

Lung function is known to differ to some extent between ethnic groups with highest values for Caucasians (11). Our case record forms did not capture ethnicity of the participants because non-Caucasians account for only a very small part of the German population. We also did not capture other known risk factors for poor lung function, such as preterm delivery/low birth weight or cardiovascular disease, specifically congestive heart failure. Based on the screening character in a pharmacy setting involving community-dwelling participants, we could only document a limited amount of parameters on each subject for pragmatic reasons, as is typical for such large-scale screening projects (20). For the same reason, our questionnaire had captured present number of daily cigarettes and presence of smoking status for less vs. more than 10 years. The dose–response relationship between smoking and COPD has largely been established for pack-years of cigarettes, but this parameter may be too complex to determine in a screening setting. Moreover, screening methods are optimized to be sensitive and not to have specificity; thus, low FEV_1_/high lung age does not establish a diagnosis but should rather be seen as stimulus to seek further clarification by a physician.

The validity of spirometry depends on several factors, including correct assessment of height, use of the device, maximum expiratory effort (effort dependence), and time between inspiration and forced expiration (time dependence). While participating pharmacy staff had received some training in the correct use of the Vitalograph, it is possible that the overall technical quality of the measurements may have been lower than, for instance, measurements in a physician office (16). We consider this, particularly a suboptimal expiratory effort, to be the most likely reason why our empirical data in non-smokers without reported airway disease for any given chronological age exhibited smaller FEV_1_ as compared to the ECSC reference equation (9). While this may have affected the absolute difference between chronological age and lung age, based on the data in Table 3, it apparently had only little impact on the assessment of factors associated with premature lung aging. Moreover, it should be considered that the classic expression of lung function as percentage predicted of FEV_1_ has lower limits of being accepted as “normal”.^2^ By contrast, the concept of lung age is not meant to be normative but rather provides a range of possible values that may or may not be considered as pathological.

A final technical consideration relates to the fact that physicians are primarily interested in effect sizes of associations. While we report such effect sizes, our regression models ranked parameters primarily by strength of association. Effect size and strength of association may not be tightly related, as a parameter with great variability in the population may exhibit a large effect size but nevertheless a rather weak association. This may explain why in our analysis, dyspnea had a considerable effect size but only a weaker association as compared to other parameters, as dyspnea is known to be a multifactorial symptom that exhibits great variability in the population. However, irrespective of the underlying cause of dyspnea, be it pulmonary, cardiac, or other, its presence has a negative impact on spirometry.

### Factors Associated with High Lung Age

The concept of lung age and its assessment by the Vitalograph have previously mainly been used in the screening for COPD (16, 17) and to motivate cessation of smoking (13, 14). However, it has been used in small populations only to provide pathophysiological information (19). Our study relates information on lung aging to possible pathophysiologically relevant factors for a cohort of more than 16,000 subjects. The respective role of such factors detected in our study is in line with general knowledge on the pathophysiology of lung disease.

Dyspnea is a symptom that can be due to a range of pulmonary, cardiac, and other conditions. It was reported by almost a third of our study participants, of which about half reported it to occur during usual daily activities, such as climbing stairs. This prevalence is similar to that reported in a recent meta-analysis of dyspnea studies in people aged 65 and older (8). While our cohort is younger than that reported by van Mourik et al., we have recruited solely pharmacy customers, which may have introduced a health bias. As in the meta-analysis by van Mourik et al., dyspnea was observed more frequently in women than men in our study. The presence of dyspnea was associated with a lung age exceeding chronological age by approximately 6 years; among subjects with dyspnea, lung age was approximately 5 years greater in those experiencing it during usual daily activities than those experiencing it only upon exertion. Overall, despite inclusion of 7.9% of patients with self-reported COPD and asthma in the model, dyspnea exhibited a stronger association with greater lung age than any other explanatory variable in our study.

Being a smoker had the second strongest association with greater lung age among candidate parameters in our study. The adverse effects of smoking on lung function are well known (21) (see text footnote 2). Being a smoker was associated with an increase in lung age by approximately 6 years in our study. Within the group of smokers, a clear dose-dependency of premature lung aging was found ranging from 6.1 to 15.4 years for those smoking 1–10 and those smoking more than 30 cigarettes per day, respectively, if compared to those smoking only occasionally. This is consistent with a smaller Vitalograph-based study, which reported greater airflow limitation in those smoking more than 20 as compared to those smoking less than 20 cigarettes per day (19). While these findings certainly are not surprising, it was proposed that confronting smokers with their greater lung age might aid cessation efforts (13, 14). Similarly, it has been proposed that calculated heart age may aid educational efforts to promote healthier life styles (15).

Self-reported COPD and asthma had somewhat weaker associations with lung aging, but with approximately 14 and 8.5 years had greater estimated effect sizes for the association with lung age than any other parameter (see “Limitation of Methods”). These effect sizes are not surprising given that the hallmarks of physiological lung aging are similar to those observed in obstructive airway disease (1, 2), i.e., that obstructive airway disease adds to the alterations that occur as part of normal aging. Thus, our data support the hypothesis that COPD can be seen as a condition of accelerated aging of the lung (12). By contrast, cough, other airway diseases (not specified as part of the study), and common cold exhibited weaker associations and only small effect sizes on lung structure and function. This is not surprising as common cold and acute cough are too short-acting to impact lung age. Among those reporting cough, the presence of expectorations and of chronic vs. acute cough did not affect lung age estimates. Presence of allergies, self-reported by a quarter of all study participants, had only very weak and inconsistent effects on lung age, possibly reflecting that the general public uses a much more lenient definition of allergy than the medical community, implying an over-reporting by medical standards.

Two parameters exhibiting moderately strong associations with greater lung age were chronological age and height. That height showed up as significant factor despite being part of the equation to calculate lung age may indicate that its association with lung age may not be as linear as the equations provided by Morris and Temple (13) would suggest. Our exploration by age deciles (Figure 2) supports this hypothesis and suggests that this may be largely driven by female participants. In fact, introducing *z*-scores in the interpretation of spirometry and abandoning fixed ranges (i.e., >80% predicted = normal) also supports this view, particularly for older women (11). That chronological age is also associated with a greater difference between lung age and chronological age may reflect that the older lung is more vulnerable to lung injury (5), and hence, for any given pathological stimulus, incurs greater damage, leading to a higher lung age estimate. In this context, one should realize that as lung age in contrast to percentage predicted FEV_1_ does not have lower limits to separate physiological from pathological values.

## Conclusion

Based on a systematic analysis of the available evidence, the U.S. Preventive Services Task Force has concluded in 2008 that a general screening for COPD based on spirometry was ineffective as it yields many false-positive cases (22). Meanwhile, many additional studies using potentially more efficient approaches have been reported, including some with handheld spirometric devices. Therefore, a recent systematic review and meta-analysis has concluded that case finding in targeted groups may be effective but stated a need for more research into the optimal identification of such targeted groups (23). Such case finding approaches apparently are well accepted by general practitioners (24). Based on a large cohort of community-dwelling subjects, our study confirms the usefulness and reliability of the Vitalograph by demonstrating that it can be used to identify and quantify risk factors associated with premature lung aging in a large population. Of note, detection of such associations is apparently robust and resistant to usual screening setting sampling errors with good performance of spirometric lung function testing by little-trained providers. Possibly, this conclusion is also applicable to other spirometry screening devices. Whereas the importance of lung age in smoking cessation is controversial, the method could be used in future epidemiological and pharmaco-epidemiological studies.

## Author Contributions

PK substantially contributed to the development of the data analysis strategy and interpretation of the data, led the search for relevant literature, has critically reviewed manuscript drafts for intellectual content, has approved the version to be published and agrees to be accountable for all aspects of the work in ensuring that questions related to the accuracy or integrity of any part of the work are appropriately investigated and resolved. TS contributed substantially to the design of the study, development of the data analysis strategy, and interpretation of the data, has critically reviewed manuscript drafts for intellectual content, has approved the version to be published, and agrees to be accountable for all aspects of the work in ensuring that questions related to the accuracy or integrity of any part of the work are appropriately investigated and resolved. TM contributed substantially to the design of the study, development of the data analysis strategy, and interpretation of the data, has critically reviewed manuscript drafts for intellectual content, has approved the version to be published, and agrees to be accountable for all aspects of the work in ensuring that questions related to the accuracy or integrity of any part of the work are appropriately investigated and resolved. HS contributed to development of the data analysis strategy and interpretation of the data, has performed the statistical analysis, has critically reviewed manuscript drafts for intellectual content, has approved the version to be published, and agrees to be accountable for all aspects of the work in ensuring that questions related to the accuracy or integrity of any part of the work are appropriately investigated and resolved. MM contributed to development of the data analysis strategy and interpretation of the data, has drafted the manuscript and critically reviewed it for intellectual content, has approved the version to be published, and agrees to be accountable for all aspects of the work in ensuring that questions related to the accuracy or integrity of any part of the work are appropriately investigated and resolved.

## Conflict of Interest Statement

In the last 5 years, Peter Kardos served on advisory boards for AstraZeneca, Chiesi, GlaxoSmithKline, Menarini, Novartis, Takeda, and Teva; received lecture fees from AstraZeneca, Boehringer Ingelheim, Cipla, Menarini, Novartis, and Teva; participated in sponsored clinical trials from AstraZeneca, Boehringer Ingelheim, and Novartis, and has been reimbursed for attending scientific symposia from AstraZeneca, Boehringer Ingelheim, Menarini, and Novartis. Tanja Schütt, Tobias Mück, and Martin C. Michel are employees of Boehringer Ingelheim; Helmut Schumacher is a former employee of Boehringer Ingelheim.

## Footnotes

^1^http://erscongress.org/programme/patient-organisation-programme.html

^2^www.goldcopd.org
